# Myelinated Afferents Are Involved in Pathology of the Spontaneous Electrical Activity and Mechanical Hyperalgesia of Myofascial Trigger Spots in Rats

**DOI:** 10.1155/2015/404971

**Published:** 2015-05-10

**Authors:** Fei Meng, Hong-You Ge, Yong-Hui Wang, Shou-Wei Yue

**Affiliations:** ^1^Department of Physical Medicine & Rehabilitation, Qilu Hospital of Shandong University, No. 107, Wenhuaxi Road, Jinan, Shandong 250012, China; ^2^Laboratory for Musculoskeletal Pain and Motor Control, Center for Sensory-Motor Interaction (SMI), Department of Health Science and Technology, Aalborg University, Fredrik Bajers Vej 7, Building D3, 9220 Aalborg, Denmark

## Abstract

Myofascial trigger points (MTrPs) are common causes for chronic pain. Myelinated afferents were considered to be related with muscular pain, and our clinical researches indicated they might participate in the pathology of MTrPs. Here, we applied myofascial trigger spots (MTrSs, equal to MTrPs in human) of rats to further investigate role of myelinated afferents. Modified pyridine-silver staining revealed more nerve endings at MTrSs than non-MTrSs (*P* < 0.01), and immunohistochemistry with Neurofilament 200 indicated more myelinated afferents existed in MTrSs (*P* < 0.01). Spontaneous electrical activity (SEA) recordings at MTrSs showed that specific block of myelinated afferents in sciatic nerve with tetrodotoxin (TTX) led to significantly decreased SEA (*P* < 0.05). Behavioral assessment showed that mechanical pain thresholds (MPTs) of MTrSs were lower than those of non-MTrSs (*P* < 0.01). Block of myelinated afferents by intramuscular TTX injection increased MPTs of MTrSs significantly (*P* < 0.01), while MPTs of non-MTrSs first decreased (*P* < 0.05) and then increased (*P* > 0.05). 30 min after the injection, MPTs at MTrSs were significantly lower than those of non-MTrSs (*P* < 0.01). Therefore, we concluded that proliferated myelinated afferents existed at MTrSs, which were closely related to pathology of SEA and mechanical hyperalgesia of MTrSs.

## 1. Introduction

Myofascial trigger points (MTrPs), which are critical for Myofascial Pain Syndrome, are hyperirritable nodules of spot tenderness in muscle taut bands (strip cords along the muscle fibers) [1]. When stimulated manually or with a needle, MTrPs induce sharp localized tenderness, spontaneous electrical activity (SEA), local twitch response (LTR) in the taut band, and a referred pain, accompanied with autonomic symptoms such as vasoconstriction and dizziness [1, 2].

SEA is the combination of endplate noise and endplate spikes with action potential [3, 4]. It consists of 2 parts: a high frequency but low amplitude persistent background current (<50 *μ*V) and a low frequency but high amplitude intermittent activity (100~700 *μ*V) [5]. Local pain intensity related to the duration and amplifies muscle cramp episodes [2]. According to the Integrated Hypothesis, muscle contractions/cramps within taut bands induced ischemia and gave rise to local pain and higher sensitivity of MTrPs [2, 6, 7]. The irritability of MTrPs affected SEA. A higher amplified SEA was usually accompanied with a lower pressure pain threshold (PPT) [8].

Myelinated afferents were considered to mediate electrically induced muscle pain [9]. Clinical researches showed sympathetic outflow modulated myelinated afferents [10] and affected pain as well as hyperalgesia at MTrPs [11]. Moreover, our previous human experiment reported, after ischemic compression block (ICB) of myelinated afferents, PPT increased while the mean amplitude and frequency of SEA decreased reversibly [12–14]. All these clinical researches indicated myelinated afferents might participate in the pathology of SEA and hyperalgesia of MTrPs.

However, current proofs were almost clinical ones and were more subjective other than objective. And some further investigations, especially invasive tests, were hardly to be applied to human beings. In order to get a more definitive conclusion about the role of myelinated afferents in peripheral changes of MTrPs with more objective and morphological proofs, we designed this animal study. Modified pyridine-silver staining method and immunohistochemistry with Neurofilament 200 (NF 200, a specific marker for myelinated afferents [15]) were firstly used to analyze nerve endings in myofascial trigger spots (MTrSs, equivalent to the human MTrPs in many aspects [16]) and non-MTrSs. SEA and behavioral changes were analyzed before and after block of myelinated afferents to explore the function of myelinated afferents in SEA and mechanical hyperalgesia of MTrSs in biceps femoris muscle of rats.

## 2. Material and Methods

### 2.1. Experimental Animals

Fifty-four adult male Wistar rats (specific pathogen free (SPF) level, weight about 300~400 g) bought from Shandong University Lab Animal Center (Jinan, China) were used in the study. All the rats were fed with food and water ad libitum in the animal house (26°C) with 12 : 12 hours of reversed light : dark cycle. This study was approved by Shandong University Animal Use Committee. Considering some gender differences, such as concentration of estrogen [17], may affect the pain pathology of rats, only male rats were used in this study.

### 2.2. Localization of MTrSs

Rats were lightly anesthetized with most of the spinal reflexes reversed and immobilized in prone position. Fur and skin covering the biceps femoris were shaved and cleared. As myofibers of taut band were stiffer than the surrounding tissues, an experienced investigator could identify the taut band (diameter of 2~3 mm) by palpating the biceps femoris. Next, squeezing the muscle along the taut band was done until an LTR existed. An LTR is brisk contraction of a group of muscle fibers in a taut band when MTrSs were stimulated. According to our experience and the current literature, usually only one most sensitive spot in the taut band can induce LTR [4], and this spot was defined as an MTrS [4, 6]. According to Integrated Hypothesis, muscle contractions/cramps within taut bands induced crisis of energy, and this vicious circle led to MTrSs finally [2, 6, 7]. As the activities of rats were not the same, not all rats exhibited MTrSs, and the active rats tend to exhibit MTrSs. Besides, individual differences should not be ignored. We chose rats with/without MTrSs as needed (described later in the Methods). Non-MTrSs were healthy spots outside the taut band without SEA or LTR.

### 2.3. Electromyography (EMG) Unit Settings for SEA Recordings and EMG-Guide Injection

A hollow-needle electrode (Ambu Neuroline Inoject, 25 × 0.30 mm, Denmark) was used as an active needle electrode inserted into MTrS region while the control needle electrode was inserted into the normal tissue, and the ground electrode was placed in the tail.

The active needle electrode was advanced about 1 mm per step until there was SEA (a high frequency but low amplitude persistent background current (<50 *μ*V) and a low frequency but high amplitude intermittent activity (100~700 *μ*V) [5]) existing on the screen and the specific electrical activity sound “Da-Da-Da” was heard. If the control electrode recorded no SEA at the same time, an MTrS was reconfirmed at the spot the active needle electrode penetrated [3, 4, 6, 18]. This spot was marked for SEA recording and injection of tetrodotoxin (TTX).

Settings of the EMG unit were as follows: the high frequency filter was 1000 Hz, while the low frequency filter was 100 Hz, the amplitude for recording was 20 *μ*V/division, and the sweep speed was 100 ms/division.

### 2.4. Section 1: Modified Pyridine-Silver Staining and Immunohistochemistry

Twenty rats were used in this section, and the MTrS/non-MTrS tissues in biceps femoris were localized and removed. 10 MTrSs and 11 non-MTrSs were used for modified pyridine-silver staining to show the nerve endings in these tissues. All the tissues were fixed in a freshly prepared fixative (consisted of ethyl alcohol, 4.5 mL, concentrated HNO_3_, 0.1 mL, and distilled water, 5 mL) for 24 hours. Then the tissue blocks were immersed in 10 mL of absolute ethyl alcohol containing 0.1 mL of ammonia solution for another 24 hours and were washed with distilled water for 30 min. After that, the blocks were stained in 2% AgNO_3_ for 3 days away from light (25°C) and were put into 10 mL of 5% formic acid containing 0.4 mg of pyrogallol for 6–24 hours [19]. After washing with distilled water, routine dehydration, transparent, paraffin immersing, and paraffin embedding of tissue blocks were carried out in sequence to prepare the paraffin blocks. Finally, both MTrSs and non-MTrSs were sectioned transversely and longitudinally at 4 *μ*m by a rotary microtome (Leica), collected, and made into paraffin sections [20]. After being dried and dewaxing, the black or dark brown nerve endings could be seen in the sections.

12 MTrSs and 8 non-MTrSs were used for the immunochemistry with NF 200 (CST) to show myelinated afferents specifically [15]. The tissue blocks were fixed in 4% paraformaldehyde overnight and were embedded in paraffin after being washed with distilled water. The paraffin blocks were cut into 4 *μ*m transverse and longitudinal sections on a rotary microtome (Leica). After that, dewaxing and antigen retrieval were carried out. After that, routine immunohistochemistry with NF 200 was carried out. NF 200 antibody was diluted to a proper concentration (1 : 100, confirmed by the preliminary tests) with phosphate-buffered saline (PBS). Peroxidase histochemistry was carried out with diaminobenzidine (DAB). Then the NF 200 positive myelinated afferents were stained brown.

In this section, for both modified pyridine-silver staining and immunochemistry, longitudinal sections were used to show location and morphology of stained fibers, while 100 nonoverlapping transverse views for MTrSs/non-MTrSs, respectively, were analyzed to get the mean number of stained fibers with light microscope equipped with a digital camera (Olympus). In order to limit the error, all measurements were performed by the same investigator.

### 2.5. Section 2: SEA Changes after Block of Myelinated Afferents in Sciatic Nerve

24 rats with MTrSs were randomly divided into TTX/isotonic saline group. After MTrS was located in the biceps femoris, a diagonal incision was made at the midpoint of ischial tuberosity and knee of the same side with MTrS. The skin, subcutaneous tissue, and fascia were dissected in sequence, and gluteus maximus was blunt dissected to expose sciatic nerve. The skin and fascia around the incision were lifted to make a bath, and artificial cerebrospinal fluid (ACSF) was used to immerse the nerve. ACSF was made up of NaCl 150 mM, KCl 5 mM, MgCl_2_ 1 mM, glucose 10 mM, CaCl_2_ 2 mM, and Tris 5 mM, and had been adjusted to pH 7.4 with HCl.

For rats in TTX group, TTX solution (1 *μ*M, Aladdin) was added into the bath to block myelinated afferents selectively [21, 22]. And for rats in isotonic saline group, isotonic saline of the same volume as TTX in the TTX group was added into the bath as the blank control. Before and ten minutes after the addition of TTX/isotonic saline, SEA was recorded stably for 3 min at MTrSs of all rats. An MTrS region was a tiny area for about 1~2 mm^2^ [23]; so after locating it, the operator had to be very careful to avoid the movement of needle electrode and make sure the electrode stays in the MTrS region all the time. According to our previous research, ischemic compression block of myelinated afferents of MTrPs resulted in a 50% decrease of the average amplitude and frequency of SEA [14]. Therefore, in this study, if the average amplitude or frequency of the high amplitude wave of SEA was 50% lower than before, it was considered as a significant change. Then the incidence rates of SEA significant changes in the two groups were analyzed. In order to limit the error, all measurements in this section were performed by the same investigator.

### 2.6. Section 3: Behavioral Assessment of Mechanical Hyperalgesia of MTrSs

Animal study revealed that stimulating masseter muscle of rats with mechanical stimulation evoked the bilateral limb nociceptive withdrawal reflex (NWR), and this model was confirmed to be an effective one to test the muscular sensitivity of lightly anaesthetized rats, and the results could been treated as “objective” ones [24–27]. Therefore, we assessed the mechanical hyperalgesia of MTrSs in the same way.

In this section, 10 rats were used. An electronic von Frey (VF) analgesiometer with a hard tip was used to stimulate the skin above the MTrSs, until ipsilateral hind-limb NWR existed. The minimum stimulus intensity that just evoked the NMR was mechanical pain threshold (MPT) which can represent the mechanical sensitivity of MTrSs/non-MTrSs [24, 25].

After MTrS was located in biceps femoris muscle of rat, a non-MTrS, a non-taut band spot (normal healthy tissue), was located in the contralateral biceps femoris muscle. First, MPTs of MTrSs and non-MTrSs were assessed, respectively. Then, 0.1 mL 1 *μ*M TTX was injected into MTrSs as well as non-MTrSs to block myelinated afferents [21]. And MPTs of MTrSs/non-MTrSs were reassessed 5 min, 15 min, and 30 min after the injection. The thresholds were measured twice with an interval of 1 min at each proper time, and the average of the two values was used as the result. In order to limit the error, all measurements in this section were performed by the same investigator. As the area of MTrS was quite small, just about 1~2 mm^2^ [23], small dose (about 0.1 mL) of liquid was used so that all of the drug could be injected [6, 18].

### 2.7. Analysis and Statistics of Data

The “a priori” sample size estimations for the rats needed in three sections were made according to the results of our preliminary experiments and the sample size estimations formulae. And the minimum of rats needed in each section was shown as follows: four rats for MTrS/non-MTrS group in modified pyridine-silver staining/IHC with NF200 in Section 1; eleven rats for TTX/isotonic saline group in Section 2; and four rats for MTrS/non-MTrS group in behavioral assessment in Section 3. Considering there might be unexpected conditions, we used a few more rats in the formal experiments. Fisher's exact test was applied to analyze the incidence rate differences of the significant SEA changes between TTX and isotonic saline groups in Section 2. All data in Sections 1 and 3 were presented as mean ± SD and analyzed with SPSS 13.0 for Windows (SPSS Inc., Chicago, IL, USA). And the results indicated that data of each variable fitted normal distribution. Student's *t*-test was used to analyze the differences of silver/NF 200 marked fibers between MTrS and non-MTrS tissues. And a two-way ANOVA followed with Student Newman-Keuls Test (SNK) was used to analyze the differences of MPTs at different time after the TTX injection between MTrS and non-MTrS. For all data, it was considered to be significant if *P* < 0.05.

## 3. Results

### 3.1. More Myelinated Afferent Nerve Endings Existed in MTrS Region

**Figure 1:**
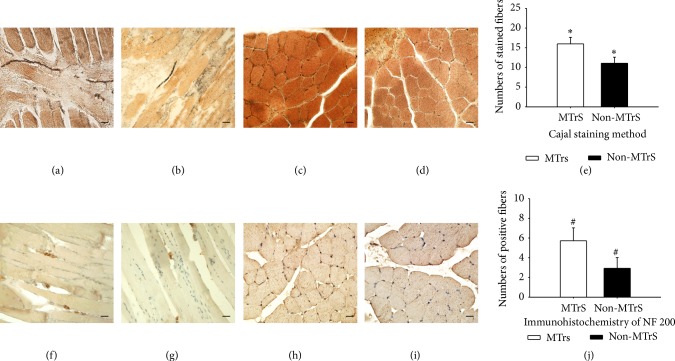
Modified pyridine-silver staining and immunohistochemistry for NF 200. (a–e) show modified pyridine-silver staining results. In the longitudinal views of MTrSs (a) and non-MTrSs (b), nerve endings are dark brown or black fibers in muscular fasciae along the muscle fibers, and in transverse views, they are dark brown or black spots between myocytes. More stained fibers exist in MTrSs (c), and fewer in non-MTrSs (d). (e) presents an illustrative diagram for modified pyridine-silver staining results, and “∗” means *P* < 0.01. (f–j) show immunohistochemistry results for NF 200. In the longitudinal views of MTrSs (f) and non-MTrSs (g), NF 200 positive myelinated afferent nerve endings are brown fibers along the muscle fibers, while in the transverse views, they are brown spots between myocytes. More positive fibers exist in MTrSs (h), and fewer exist in non-MTrSs (i). (j) presents an illustrative diagram for immunohistochemistry results, and “#” means *P* < 0.01. Scale bar = 20 *μ*m.

In Section 1, by applying modified pyridine-silver staining method, silver was reduced into dark brown or black precipitation to present nerve endings in biceps femoris muscle. Light microscopy revealed silver stained fibers in muscular fasciae along the muscle fibers in both MTrSs and non-MTrSs, and MTrSs contained significantly more nerve endings (15.97 ± 1.666) than non-MTrSs (11.06 ± 1.543) (*P* < 0.01) (see Figures 1(a)–1(e)). Immunohistochemistry with NF 200 showed brown positive myelinated afferent fibers in muscular fasciae along the muscle fibers in MTrSs as well as non-MTrSs, and MTrSs contained more myelinated afferent nerve endings (5.720 ± 1.311) than non-MTrSs (2.930 ± 1.085), and the difference was significant (*P* < 0.01) (see Figures 1(f)–1(j)).

### 3.2. SEA at MTrS Region Decreased Significantly after Specific Block of Myelinated Afferents in Sciatic Nerve

**Figure 2:**
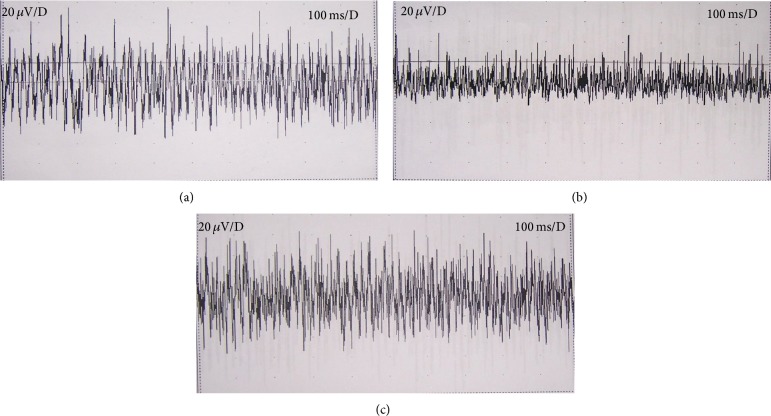
SEA recorded at MTrSs before and after adding TTX/isotonic saline in to the bath. (a) indicates SEA at MTrSs before the addition of TTX/isotonic saline; (b) indicates both amplitude and frequency of SEA decreased after the addition of TTX; and (c) indicates SEA did not change obviously after the addition of isotonic saline.

For Section 2, in the TTX group, 7 rats showed significantly decreased SEA, while the other 5 rats had decreased, but not significantly changed, SEA after TTX addition. In the isotonic saline group, 2 rats had significantly decreased SEA after the addition of isotonic saline and the rest of the rats just had no significant changes (see Figure 2). Incidence rates of SEA significant changes between the two groups were significantly different (*P* = 0.0447).

### 3.3. MPT of MTrS Was Lower Than That of Non-MTrS, and MTrS Was More Sensitive to Block of Myelinated Afferents

For Section 3, a two-way ANOVA revealed, 5, 15, and 30 min after the block, there were significant differences between MPTs of MTrSs and non-MTrSs, and there was significant interaction between these two factors (5 min after the block: *F* = 12.141, *P* < 0.01; 15 min after the block: *F* = 96.128, *P* < 0.01; 30 min after the block: *F* = 6.913, *P* < 0.01).

**Figure 3:**
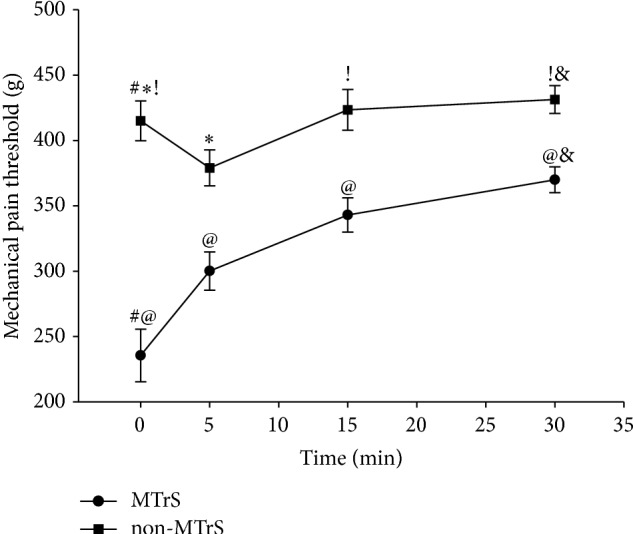
Mechanical pain thresholds at MTrSs/non-MTrSs that evoked ipsilateral hind-limb nociceptive withdrawal reflex (NWR). “#” represents, before the TTX injection, MPTs at MTrSs were significantly lower than those at non-MTrSs (*P* < 0.01). “@” represents, 5 min, 15 min, and 30 min after the TTX injection, MPTs at MTrSs increased significantly (*P* < 0.01). “∗” represents, 5 min after the TTX injection, MPTs at non-MTrSs decreased (*P* < 0.05). “!” represents, 15 min and 30 min after the TTX injection, MPTs at non-MTrSs were higher than those at non-MTrSs before injection, but the differences did not reach the significant level (*P* > 0.05). “&” represents, 30 min after TTX injection, MPTs at MTrSs were significantly lower than those at non-MTrSs (*P* < 0.01).

Prior to the injection of TTX, MPTs in MTrSs were significantly lower than those in non-MTrSs (*q* = 12.438, *P* < 0.01). And MPTs at MTrSs increased significantly 5, 15, and 30 min after TTX injection (5 min after the block: *q* = 4.476, *P* < 0.01; 15 min after the block: *q* = 7.504, *P* < 0.01; 30 min after the block: *q* = 9.313, *P* < 0.01). For non-MTrSs, MPTs decreased significantly 5 min after the injection (*q* = 3.569, *P* < 0.05), while 15 min/30 min after the TTX injection, MPTs increased when compared with those prior to the injection, but the differences were not significant (15 min after the block: *q* = 1.129, *P* > 0.05; 30 min after the block: *q* = 0.547, *P* > 0.05). Thirty minutes after TTX injection, MPTs of MTrSs were significantly lower than those of non-MTrSs (*q* = 4.255, *P* < 0.01) (see Figure 3 and Table 1).

## 4. Discussion

This study further investigated the function of myelinated afferents in pathology of MTrSs. We found more nerve endings, especially more myelinated afferents, existed in MTrS regions than normal tissue. And specific block of myelinated afferents with TTX resulted in decreased SEA as well as higher MPTs of MTrSs. Thus, we concluded proliferated myelinated afferents were closely related with SEA and mechanical hyperalgesia of MTrSs.

Modified pyridine-silver staining method was used to indicate sensory nerve endings in skeletal muscles, and these nerve endings were stained as dark brown or black [19]. In this current study, we applied this method to show nerve endings in MTrS regions for the first time and found there were more nerve endings in MTrS region than non-MTrS region. Previous study revealed similar results by applying iron deposition method, and they indicated these nerve endings were nociceptive ones [28, 29]. Additionally, in this study, immunohistochemistry for NF 200 was performed [15] and revealed more myelinated afferents in MTrS region than normal tissues.

The reason for proliferated myelinated afferents was not clear yet. It was reported that, after nerve injury, membrane properties of myelinated afferents changed which induced sprout of axon endings [30]. We thought local environment changes at MTrS regions, including changes of electrolytes, pH, muscle metabolites, inflammatory mediators, and neurotransmitters [3, 7], might induce the sprout of myelinated afferents as well and lead to increased number of them.

In order to investigate the function of myelinated afferents, we selected TTX to block them specifically [21, 22, 31]. TTX is a neurotoxin, which can block the rapid sodium channels in fast conducted myelinated afferents with little effect to slow sodium channels in unmyelinated afferents [32]. After blocking myelinated afferents, SEA and MPT of MTrSs changed accordingly, which indicated the relations between myelinated afferents and MTrSs. And our previous clinical study supported this; ischemic compression blockage (ICB) of myelinated afferents changed SEA and pressure pain threshold (PPT) as well as pressure threshold for eliciting referred pain (PTRP) [13, 14].

SEA is one of the characteristics of MTrSs. It is a combination of endplate noise and endplate spikes with action potential [3, 4, 23, 33] and can be recorded at MTrPs/MTrSs with EMG equipment. Nociceptive stimuli, such as inflammation, induced higher sensitivity of myelinated afferents and raised SEA [34]. TTX blocked the action activity of fast conducted myelinated afferents [32], and then amplitude and frequency of SEA decreased.

As no criterion about SEA changes was generally recognized, in Section 2, we chose 50% decrease of amplitude or frequency of the high amplitude wave as a significant change (according to our previous clinical results [14]). Any SEA change that did not reach this level was considered to be no significant one, even if their amplitude/frequency did decrease.

And it was worth noticing that although incidence rates of SEA significant changes caused by TTX/isotonic saline were significantly different (*P* < 0.05), there were still 5 rats that had decreased but not significantly changed SEA after applying TTX. We inferred the reasons might be as follows. Individual differences of rats, such as numbers of sodium channels on myelinated afferents, led to different sensitivity to TTX [35, 36]. And myelinated afferents of these 5 rats might be blocked to a lesser extent when compared with the other 7 rats in TTX group; thus, their amplitude or frequency of SEA decreased, but did not reach significant level according to our criterion [14]. In addition, C fibers and sympathetic nerves, which were less sensitive to TTX [32, 37], were inferred to innervate muscle spindles and modify SEA of MTrSs as well [38–41], and they might influence the results. Besides, our criterion for significant changes might be a little too strict, and these not significantly changed SEAs in the 5 rats were possible to be significant ones according to a looser criterion. Further studies were needed to find an improved criterion and better understand SEA.

Noxious cutaneous stimulations elicit nociceptive withdrawal reflex (NWR), which protects the subject from nociceptive damage. NWR is a spinal polysynaptic reflex, and it has been used to investigate pain processing as well as pain pathways at spinal levels [26]. Moreover, the results of behavior with NWR can be used as an “objective” measure of experimental pain [24, 25, 27]. Therefore, we assessed mechanical hyperalgesia of MTrSs/non-MTrSs in the same way. MPT at non-MTrS regions increased, 15 min or 30 min after the TTX injection, but not significantly, while that of MTrS regions increased significantly at any time after the injection. It seemed that block of myelinated afferents affected the mechanical hyperalgesia of both MTrSs and non-MTrSs, and MTrSs were more sensitive to the block of myelinated afferents. An explanation for this was that there were more myelinated afferents existing in MTrSs than in non-MTrSs, which could be proved by Section 1 of this study.

Besides, 30 min after the injection, although the MPT of MTrS was obviously increased, it was still lower than that of non-MTrS. We inferred C fibers and sympathetic nerves were responsible for this. C fibers participated in the conduction of nociceptive stimuli, increased the sensitivity of MTrSs [29, 38–40], and led to decreased MPT of MTrSs [8]. Moreover, sympathetic nerves were indicated to modify MTrS related pain via sympathetic-sensory interactions [10]. Both of them were much less sensitive to TTX than myelinated afferents [32, 37], and therefore, they might be potential reasons for the MPT differences between MTrS and non-MTrS after blocking myelinated afferents. Additionally, MPT of non-MTrS decreased 5 min after the TTX injection, and a likely explanation was that the injection operation might stimulate C fibers.

Although this study demonstrated myelinated afferents affected SEA and mechanical hyperalgesia of MTrSs, the mechanism was still mysterious. We hypothesized the reactions between myelinated afferents and sensitization (both central and peripheral) of MTrSs [7] were responsible for this process. We found that muscle fibers in MTrSs were especially prone to fatigue [42, 43], and these fatigue muscle fibers could enhance input impulses of myelinated afferents [44]. According to the Integrated Trigger Point Hypothesis [1, 2, 6, 7], muscle contractions/cramps within taut bands led to ischemia and energy crisis and induced release of neural active substances and metabolites and resulted in higher sensitivity of MTrSs [2, 6, 7] and higher sensitivity and proliferation of myelinated afferent terminals [1, 7, 30]. The irritability of MTrS is related to SEA [8], while proliferated sensitive myelinated afferents conducted more peripheral nociceptive stimuli to the central nerve system, participated in the development of central sensitization by sprout and phenotypic switch, and induced hyperalgesia of MTrSs [45–47]. In addition, increased sympathetic activity and pain intensity were reported at MTrPs, and the sympathetic nerves in muscle spindles were consisted to be related with sensitized myelinated afferents and gave rise to lower pain threshold, higher pain intensity, and increased SEA at MTrPs [10, 11, 13, 14, 41].

The mechanism of MTrSs is mysterious, and in this study, we found proliferated myelinated afferents modified SEA and mechanical hyperalgesia of MTrSs and proposed a hypothesis of the modulatory mechanism. More studies, such as investigating central sensitization with immunohistochemistry about the nociceptive related receptors in spinal cord, afferent nerve tracing to show their connections and conduction, were already in operation to reveal the exact mechanism.

## 5. Conclusion

This study demonstrated that MTrSs contained more myelinated afferents than normal healthy tissues, which were associated with pathology of SEA and mechanical hyperalgesia of MTrSs. Nevertheless, how myelinated afferents affect SEA and mechanical hyperalgesia was still uncertain. We hypothesized myelinated afferents affected SEA and mechanical hyperalgesia of MTrSs through central and peripheral sensitization. More studies were already in operation to reveal the exact mechanism.

## Figures and Tables

**Table 1:** Mechanical pain thresholds at MTrSs/non-MTrSs at different time before and after TTX injection.

Time (min)	MTrSs		Non-MTrSs	
	Mean (g)	SD (g)	Mean (g)	SD (g)
0	235.50^#@^	20.05	415.00^#∗!^	15.22
5	300.10^@^	14.63	379.00^∗^	13.74
15	343.00^@^	13.12	423.40^!^	15.63
30	369.90^@&^	9.94	431.30^!&^	10.70

“#” represents, before the TTX injection, MPTs at MTrSs were significantly lower than those at non-MTrSs (*P* < 0.01). “@” represents, 5 min, 15 min, and 30 min after the TTX injection, MPTs at MTrSs increased significantly (*P* < 0.01). “∗” represents, 5 min after the TTX injection, MPTs at non-MTrSs were lower than those before the injection (*P* < 0.05). “!” represents, 15 min and 30 min after the TTX injection, MPTs at non-MTrSs were higher than those at non-MTrSs before injection, but the differences did not reach the significant level (*P* > 0.05). “&” represents, 30 min after TTX injection, MPTs at MTrSs were significantly lower than those at non-MTrSs (*P* < 0.01).
